# Investigating Stroke Effects on Respiratory Parameters Using a Wearable Device: A Pilot Study on Hemiplegic Patients

**DOI:** 10.3390/s22176708

**Published:** 2022-09-05

**Authors:** Joshua Di Tocco, Daniela Lo Presti, Martina Zaltieri, Marco Bravi, Michelangelo Morrone, Silvia Sterzi, Emiliano Schena, Carlo Massaroni

**Affiliations:** 1 Unit of Measurements and Biomedical Instrumentation, Università Campus Bio-Medico di Roma, 00128 Rome, Italy; 2 Unit of Physical and Rehabilitative Medicine, Università Campus Bio-Medico di Roma, 00128 Rome, Italy

**Keywords:** hemiplegic patient, physiological monitoring, piezoresistive sensors, respiratory monitoring, strain sensor, wearable device

## Abstract

Quantitatively assessing personal health status is gaining increasing attention due to the improvement of diagnostic technology and the increasing occurrence of chronic pathologies. Monitoring physiological parameters allows for retrieving a general overview of the personal health status. Respiratory activity can provide relevant information, especially when pathologies affect the muscles and organs involved in breathing. Among many technologies, wearables may represent a valid solution for continuous and remote monitoring of respiratory activity, thus reducing healthcare costs. The most popular wearables used in this arena are based on detecting the breathing-induced movement of the chest wall. Therefore, their use in patients with impaired chest wall motion and abnormal respiratory kinematics can be challenging, but literature is still in its infancy. This study investigates the performance of a custom wearable device for respiratory monitoring in post-stroke patients. We tested the device on six hemiplegic patients under different respiratory regimes. The estimated respiratory parameters (i.e., respiratory frequency and the timing of the respiratory phase) demonstrated good agreement with the ones provided by a gold standard device. The promising results of this pilot study encourage the exploitation of wearables on these patients that may strongly impact the treatment of chronic diseases, such as hemiplegia.

## 1. Introduction

Monitoring personal health status has always been relevant for each individual to quantitatively assess and try to prevent, cure, or mitigate any illness. It is all the more so in the presence of worldwide diseases, which find optimized outcomes relying on personal health status information provided by people around the world [1,2].

The first understanding of a person’s health status can be achieved by measuring multiple physiological parameters (e.g., heart rate, respiratory rate, arterial pressure). Particularly, when affected by respiratory diseases or chronic pathologies (i.e., post-stroke patients), which require significant attention and a long time for being cured or in some cases last a lifetime. Obtaining quantitative information on how the patient condition is evolving is pivotal for assigning the correct rehabilitation therapy and improving its outcomes. Thus, the continuous monitoring of respiratory parameters can provide an important contribution to tailor personalized patients’ therapy [3,4].

To this purpose, a variety of technologies can be used, characterized by different performance, cost, affordance, and ease of use. The gold standard systems for the estimation of respiratory parameters are spirometers [5,6,7] and motion-capture systems [8,9,10]. In particular, a specific motion analysis system (i.e., opto-electronic plethysmography, OEP) allows tracking the 3D trajectories of photo-reflective markers positioned on the chest wall following a specific marker protocol [11,12], providing a deeper understanding of the respiratory kinematics and the possibility to analyze single chest wall compartments with excellent performance.

Although OEP has great performance, it requires a high cost for its use, a structured environment, a calibration to retrieve the respiratory data, and qualified personnel to prepare the subject for the examination and post-process the raw data. In addition, mostly the calibration and the preparation of the subject are time-consuming activities. On the other hand, spirometers provide a more portable solution for assessing respiratory parameters, but they cannot monitor the respiratory activity of single chest wall compartments, still require qualified personnel, and provide discomfort during prolonged use due to the use of the mouthpiece.

To cope with these aspects, wearable systems result to be a valuable alternative due to their cost-effectiveness, ease of use, and performances [13,14,15]. Moreover, in the framework of telemedicine and personalized medicine, they enable continuous monitoring of the patient, reducing healthcare costs, and mental and physical stress undergone when reaching the clinic in a day-to-day scenario. These devices are able to estimate respiratory activity by measuring different parameters (e.g., chest wall motion, respiratory sound, respiratory airflow) [16,17,18]. Among these, wearables based on measuring the chest wall motion can be developed using different sensing technologies, which are fiber Bragg grating (FBG) [19,20,21,22], inductive [23,24,25,26], capacitive [27,28,29,30], and conductive sensors [31,32,33,34].

These systems have been tested in many scenarios (e.g., sports activities, occupational settings and laboratory settings), demonstrating good results in healthy subjects [27,28,31,35,36]. However, their performance may dramatically change in post-stroke patients, since hemiplegia affects the function of chest wall muscles; thus, the entire respiratory kinematics [37,38,39]. This concern is worthy of investigation, as testified by several studies performed with OEP [40,41,42], but the assessment of wearables in patients with abnormal respiratory kinematics is still in its infancy.

In this study, we investigate the capability of a multi-sensor wearable device (WD) to monitor the respiratory parameters in six post-stroke patients, and we compared its performance with an OEP used as a gold standard. We also analyzed how the system performances are influenced by the sensors’ position (e.g., placed on the healthy side and affected side), showing the relevant influence of this choice, especially in the tachypnea regime.

## 2. Materials and Methods

In this section, we present the sensing technology used to instrument the proposed wearable device for monitoring respiratory activity, the experimental protocol performed during the pilot study on hemiplegic patients, and the data analysis performed on the retrieved data.

### 2.1. Wearable Device

**Figure 1:**
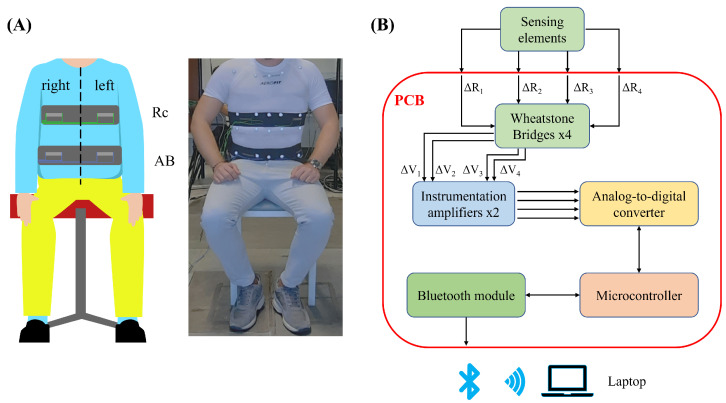
(**A**) Graphical representation of the position of the elastic bands on the chest wall and of the position of each sensor on the elastic band, and (**B**) measurement chain diagram representing all the WD components.

The WD consists of two elastic bands integrating two sensing elements each. This multi-sensor design has been chosen to enable the monitoring of the respiratory activity in the different chest wall compartments by using the presence of the two bands; in addition, for each band, there is a sensing element placed in the affected side and one sensing element placed in the healthy one. The sensing elements are made of an elastic piezoresistive textile composed of 89% silver and 11% spandex, which changes its resistance according to the applied strain. In this case, the strain is provided to the elements from the cyclical expansion and contraction of the rib cage during the respiratory activity. The elements have been hand cut in rectangles from an A4 sheet of material using scissors (nominal dimensions, length 50 mm, and width 10 mm). To integrate the elements on the elastic bands, cotton threads have been used to sew the shorter extremities and provide anchoring points for the sensing element. The elements have been positioned on the bands in a way that allows each sensor to be placed on each side (i.e., right, and left) of the correspondent chest wall compartment (i.e., rib cage—Rc-, and abdomen—AB-). Figure 1A shows a graphical representation of the developed WD highlighting the sensors’ positioning on the chest wall. To enable the use of the WD from multiple subjects, Velcro has been used as a fastening method to make the device adaptable to different anthropometries. To retrieve the respiratory signal from the sensing elements, a custom-printed circuit board (PCB) has been developed. This board consisted of four Wheatstone bridges used to transduce the resistance of the sensing elements into voltage, two instrumentation amplifiers (AD8426, by Analog Devices, Norwood, MA, USA) one for each pair of Wheatstone bridges, a 12-bit Analog-to-Digital Converter (MAX 1237, by Maxim Integrated, San Jose, CA, USA), a microcontroller (PIC18F46J50 I-PT by Microchip, Chandler, AZ, USA), and a Bluetooth module (SPBT2632C2A by ST Microelectronics, Geneva, Switzerland). The WD’s measurement chain is shown in Figure 1B. To connect the sensing elements to the electronics, electrical copper wires were fixed to the short extremities of the sensing elements. Finally, to collect the data wirelessly from the WD, a laptop running a custom data collection algorithm in MATLAB was used. All the retrieved data were collected at 100 Hz.

### 2.2. Reference Device

**Figure 2:**
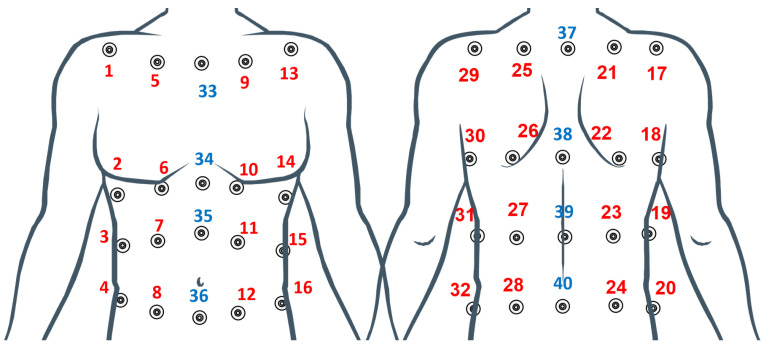
Marker positioning on the back and front side of the chest wall surface. The chest wall is subdivided into two main compartments: Rc and AB. Rc extends from the clavicles to the lower costal margin line of markers. AB covers the caudal parts of the frontal chest, from the lower costal margin to the level of the anterior superior iliac crest. Four posterior horizontal rows (between C7 and the posterior axillary lines) contribute to the coverage of the back. The blue markers represent the additional 8 markers to the 32 marker protocol.

**Figure 3:**
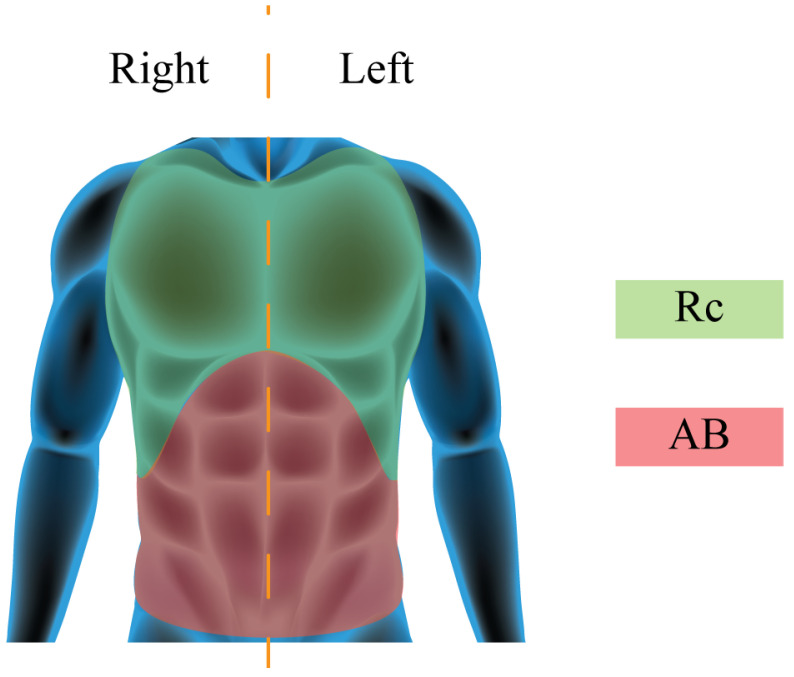
Graphical representation of the chest wall subdivision into 2 main compartments. In green the Rib cage, Rc, and in red the abdomen, AB.

A motion capture system (Smart-D, BTS Bioengineering Corp., Milan, Italy), hereinafter referred to as a mocap, was used as a reference device for detecting chest wall displacements and compartmental breathing volumes. To calculate the breathing volumes from chest wall displacements, a chest wall model must be developed. Different marker protocols have been used in previous studies based on 89, 86, 32 and 30 markers. The 89 marker model seems to allow a more accurate reconstruction of the chest wall surface and is therefore considered the gold standard. However, the high number of markers required can affect the reproducibility of measurements, and markers placing, data collection, and post-processing of the marker data can be challenging and time-consuming [43]. Recently, the 32 marker protocol reported in [44] has been demonstrated to be sufficiently accurate to determine rib cage and abdomen compartmental volumes and thoraco-abdominal motion pattern (in terms of compartmental percentage contributions, and coordination between compartments). In this paper we adopted a 40 marker protocol to better differentiate the left and right side of the chest wall needed for the specific application (hemilateral kinematics assessment). The 40 markers are positioned according to Figure 2 and the chest wall Rc and AB compartments are shown in Figure 3.

### 2.3. Experimental Protocol

**Figure 4:**
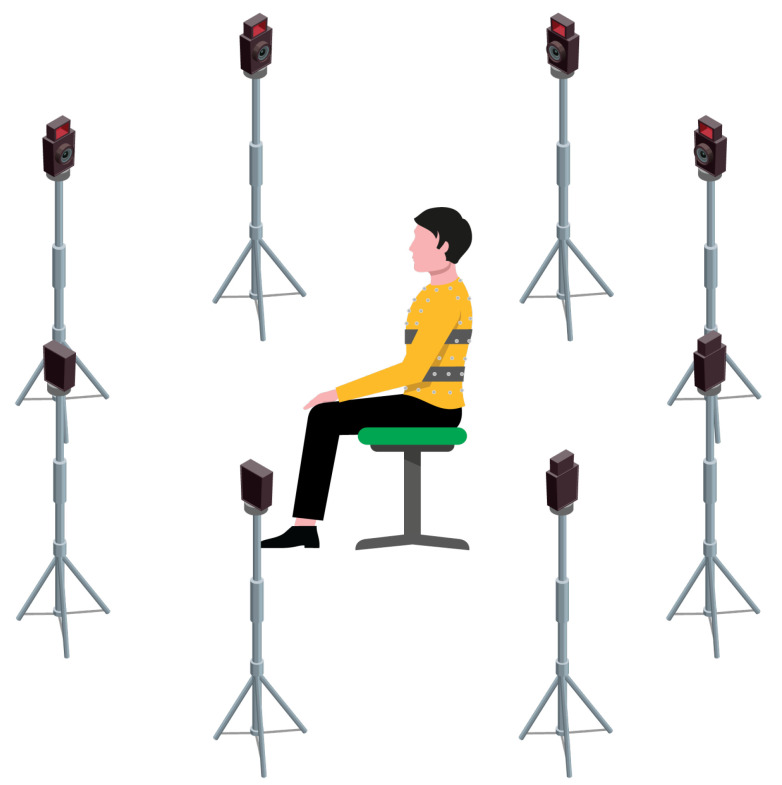
Schematic representation of the experimental setup showing the subject sitting on a stool in the center of the room wearing the WD and the photoreflective markers (grey spheres on the chest wall) surrounded by the mocap cameras.

Six post-stroke patients (4 male, 2 female, average age 50.5 years) were enrolled for this study. Inclusion criteria were as follows: Hemiplegic subjects with ischemic or hemorrhagic injury, with diagnosis confirmed by brain imaging systems (MRI, CT); subjects who retain sufficient cognitive and speech functions to follow the instructions given by the physician and therapist; subjects capable of independently maintaining orthostatic position. The severity of the plegia has been assessed using the Fugl-Meyer score. Details of patients’ anthropometric measurements, affected side and plegia severity are reported in Table 1. All patients have been asked to wear the wearable device (one band on between the first and second marker line in Figure 2, and one between the third and fourth marker line in Figure 2) and the photoreflective markers and to perform the following trials while sitting on a stool (see Figure 4):

- *Eupnea trial*: self-paced eupnea, 10 s apnea, approximately 60 s of self-paced eupnea, and 10 s apnea;
- *Tachypnea trial*: self-paced breathing, 10 s apnea, approximately 40 s of self-paced tachypnea, and 10 s apnea.

The experimental trials have been conducted in accordance with the guidelines of the Declaration of Helsinki and approved by the Institutional Ethics Committee of Università Campus Bio-Medico di Roma (27/18 OSS ComEt UCBM). Informed consent was obtained from all subjects involved in the study.

### 2.4. Data Analysis

Data analysis has been conducted by firstly preprocessing the collected data and then using two different methods to assess the performances of the devices (i.e., frequency-based and time-based). The WD performances were analysed in terms of respiratory frequency and timing of the respiratory phases by comparing the values estimated by the proposed system with those provided by the mocap.

#### 2.4.1. Preprocessing

The WD data have been preprocessed by a filtering stage characterized by a 3rd order bandpass Butterworth digital filter with a low cutoff frequency of 0.05 Hz and a high cutoff frequency of 2 Hz. Left and right chest wall compartment respiratory signals have been obtained by summing the left Rc and AB signals, and by summing the right Rc and AB signals, respectively. The total chest respiratory signal was obtained by summing the left and right chest wall compartment respiratory signals.

The mocap data have been preprocessed by applying a previously developed geometric model implemented using a custom MATLAB code to compute the chest volume from the 3D marker coordinates [45]. The coordinates of the body landmarks were used as a starting point for the geometric model to compute volume. Each compartment consists of eight markers: four on the front and four on the back of the chest (some compartments share the same markers). The eight markers identify six tetrahedrons [45,46]. The volume of each tetrahedron is computed starting from the coordinates of its vertices. By considering a generic tetrahedron with vertices $P_{1}$, $P_{2}$, $P_{3}$, $P_{4}$ the volume enclosed can be calculated, as in the following equation.

(1) $$V=\frac{1}{6}\left|det(V_{1},V_{2},V_{3}))\right|=\frac{1}{6}\left|det\left[\begin{array}{cccc} 1 & x_{p_{1}} & y_{p_{1}} & z_{p_{1}}\\ 1 & x_{p_{2}} & y_{p_{2}} & z_{p_{2}}\\ 1 & x_{p_{3}} & y_{p_{3}} & z_{p_{3}}\\ 1 & x_{p_{4}} & y_{p_{4}} & z_{p_{4}} \end{array}\right]\right|$$

where $V_{1}$ = $P_{2}-P_{1}$, $V_{2}$ = $P_{3}-P_{2}$, and $V_{3}$ = $P_{4}-P_{3}$. The sum of all tetrahedral equals the total chest volume, and the sum of the tetrahedral volumes in each compartment adds up to the compartmental volume.

To synchronize the two devices, the end of the first 10 s apnea (i.e., first minimum after the apnea) and the beginning of the last 10 s apnea (i.e., first minimum before the apnea) have been used as synchronizing points.

#### 2.4.2. Frequency-Based Method

To compare the performances of the two devices in estimating the average respiratory frequency, the error (err) and the mean absolute error (MAE) have been calculated. The average respiratory frequency has been calculated by performing the Power Spectral Density (PSD) using the pwelch estimator. This analysis has been performed on the total respiratory signal (i.e., sum of all compartments), on the affected and healthy sides (i.e., sum of the affected and healthy compartments according to Table 1). Then, by multiplying the frequency value corresponding to the maximum peak of the PSD by 60, the number of breaths per minute (i.e., average respiratory frequency) expressed in bpm (breaths per minute) was obtained. Finally, the err value has been calculated as in the following equation.

(2) $$err_{xx}\left(i\right)=f_{xx}^{m}\left(i\right)-f_{xx}^{p}\left(i\right)$$

where $f_{xx}^{m}$ represents the average respiratory frequency estimated by the mocap and $f_{xx}^{p}$ represents the average respiratory frequency estimated by the WD. The argument *i* represents the *i*-th patient and the subscript xx represents the compartments involved into the calculation, which are tt for the total chest wall, hh for the healthy side, and aa for the affected side.

The MAE has been calculated from the err as in the following equation:

(3) $$MAE=\frac{\sum_{i=1}^{N}\left|err_{xx}\left(i\right)\right|}{N}$$

where $err_{xx}\left(i\right)$ represents the average estimation error calculated previously for the *i*-th patient, and *N* represents the number of patients.

#### 2.4.3. Time-Based Method

**Figure 5:**
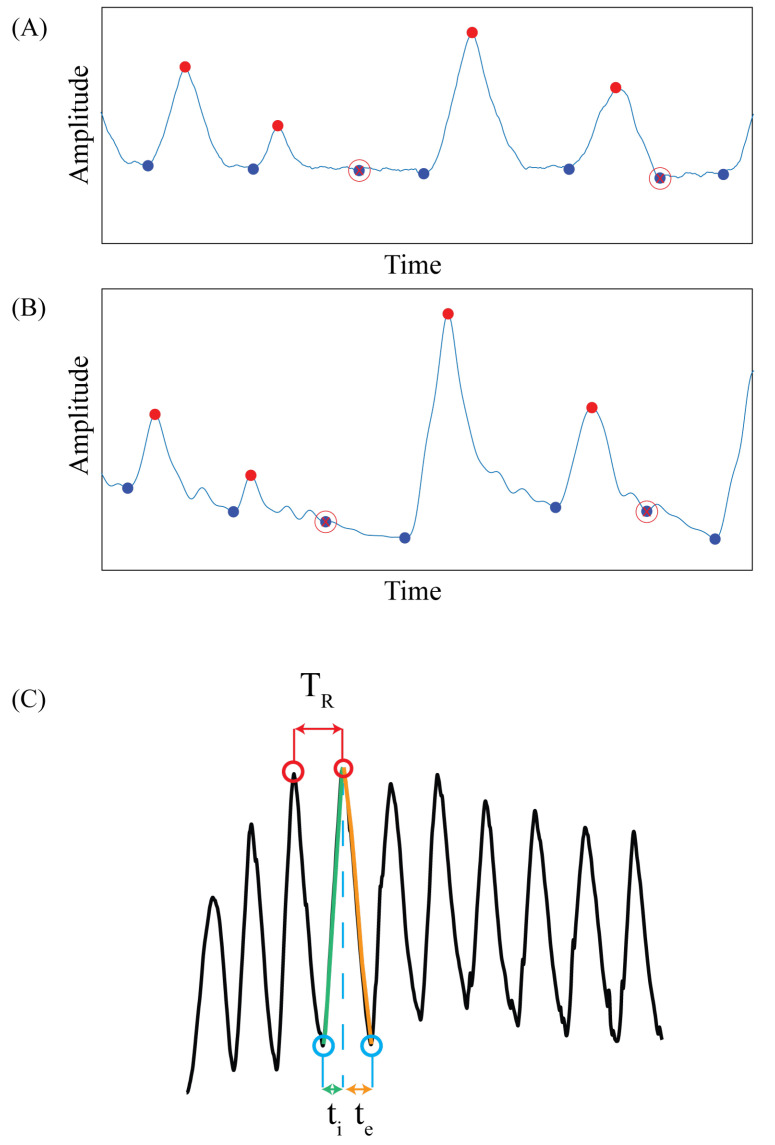
Example of peak misdetection performed by the algorithm on mocap signal (**A**), and on the wearable device (**B**). The maximum peaks are highlighted with the red dots and the minima peaks are highlighted with the blue dots.The misdetected peaks are highlighted by the circled red x. (**C**) shows the identification of the respiratory period ($T_{R}$) from which the $f_{r}$ has been calculated, the inspiratory time ($t_{i}$), and the expiratory time ($t_{e}$) on the respiratory signal.

In this analysis, temporal parameters associated to the respiratory activity have been calculated. In particular, breath-by-breath respiratory frequency ($f_{r}$), inspiratory time ($t_{i}$), and expiratory time ($t_{e}$). To identify each breath and its phases (i.e., inspiration and expiration) from the respiratory signal, inspiratory (maxima) and expiratory (minima) peaks have been identified by performing an automatic peak detection on the normalized signals. This peak detection algorithm is based on two thresholds: one is a temporal threshold, defining the minimum distance between two consecutive peaks, and an amplitude threshold, defining the minimum amplitude of the normalized signal over which the peak has to be detected. Due to this automated peak detection and thresholding, in some cases especially at the end of the expiratory phase or in presence of abnormal signals (e.g., motion artifacts, irregular respiratory pattern), misdetected peaks (i.e., <2% of the total peaks) have been corrected after visual inspection of the results of the automatic peak detection. Figure 5A and Figure 5B shows an example of the misdetected peaks on the mocap and WD, respectively.

$f_{r}$ has been calculated as the reciprocal of the time difference between consecutive maxima (i.e., respiratory period, T_R_), $t_{i}$, and $t_{e}$ have been calculated as the time difference between a minimum and the following maximum, and the difference between a maximum and the following minimum, respectively. Figure 5C shows an example of peak detection performed on a respiratory signal and how the temporal parameters have been calculated. To compare the obtained respiratory parameters, Bland-Altman (BA) analysis has been performed separately for each parameters and for each compartment comparison. According to [47] the following parameters have been calculated:

- *Mean of Differences* (MOD): difference between the estimated p by the mocap and the one estimated by the WD;
- *Limits of Agreement* (LOA): MOD ± (1.96 · SD (p));
- *LOA amplitude* (LOA amp): 2 · (1.96 · SD (p)).

where SD represents the standard deviation, and p is either $f_{r}$, $t_{i}$ or $t_{e}$ estimated in the various chest wall compartments.

Finally, the MAE has been calculated as in Equation (3), where $err_{xx}\left(i\right)$ represents the error committed by the WD in estimating $f_{r}$, $t_{i}$, and $t_{e}$ on the i-th breath, and *N* represents the total number of breaths equal to the sum of the number of breaths of each subject. The MAE has been calculated separately for the eupnea and tachypnea trials.

## 3. Results

In this section, the results obtained from the frequency- and time-based methods are reported.

### 3.1. Frequency-Based Method

When comparing the performances of the WD with the mocap in estimating the respiratory frequency in the different compartments (i.e., total chest wall, healthy side, affected side), promising results were obtained. Indeed, in the eupnea regime the err was always lower than 1.1 bpm and the maximum MAE was 0.34 bpm. These worst cases were found in the hh and aa comparison, while better results were recorded in tt. In tachypnea, the err was always ∼ 0 bpm, except in patient 1 in the hh comparison, where the err was equal to 2.01 bpm, and in patients 2 and 3 in the aa comparison the err was 2.01 bpm and 5.99 bpm, respectively. It is worth noting that the resolution in the frequency domain is 0.01 Hz; this motivates the null difference in a large part of the trials. The MAE showed values lower than 1.35 bpm. Table 2 reports all the err and MAE values calculated for all patients in all trials.

### 3.2. Time-Based Method

**Figure 6:**
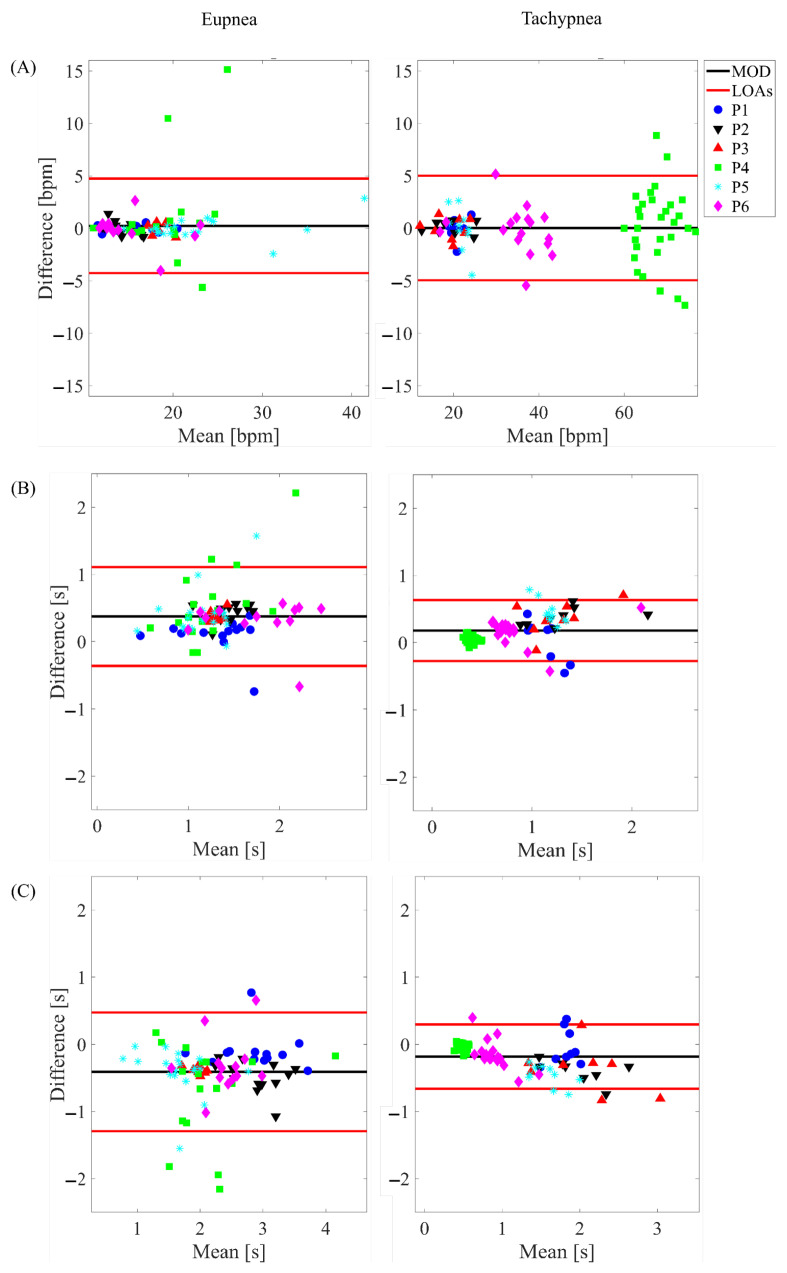
Bland-Altman plots depicting the estimated (**A**) $f_{r}$, (**B**) $t_{i}$ and (**C**) $t_{e}$ on the total respiratory signal in eupnea and tachypnea trials.

**Figure 7:**
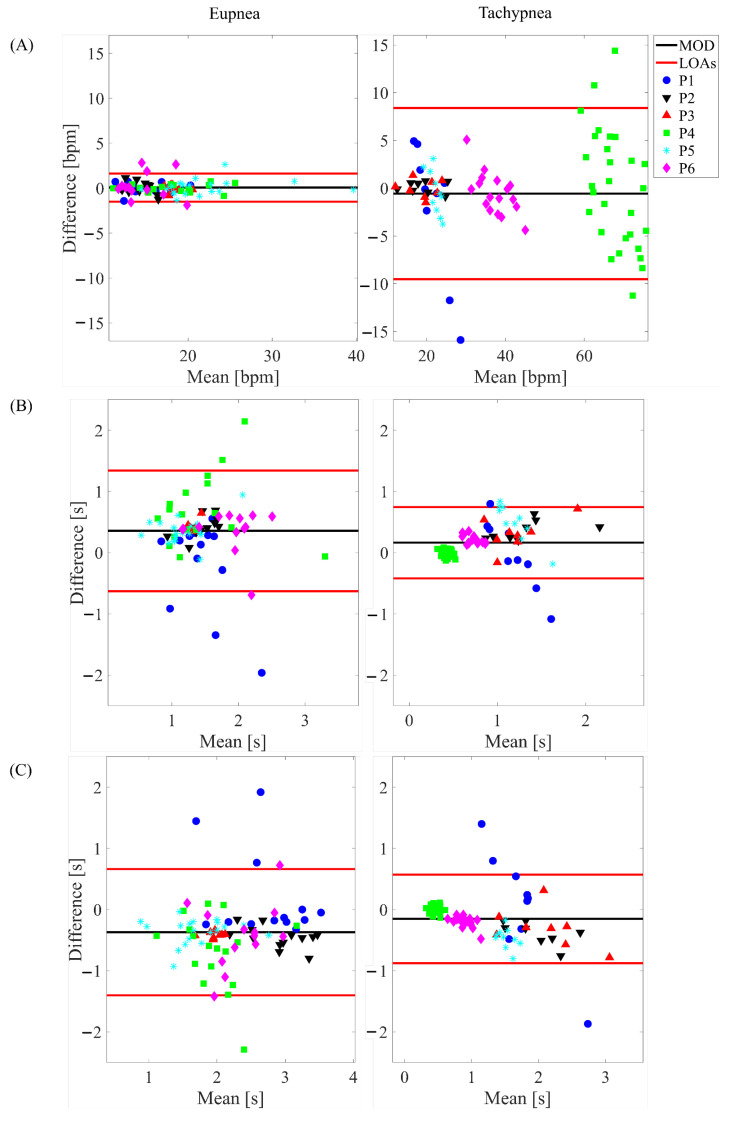
Bland-Altman plots depicting the estimated (**A**) $f_{r}$, (**B**) $t_{i}$ and (**C**) $t_{e}$ on the respiratory signal retrieved from the healthy side in eupnea and tachypnea trials.

**Figure 8:**
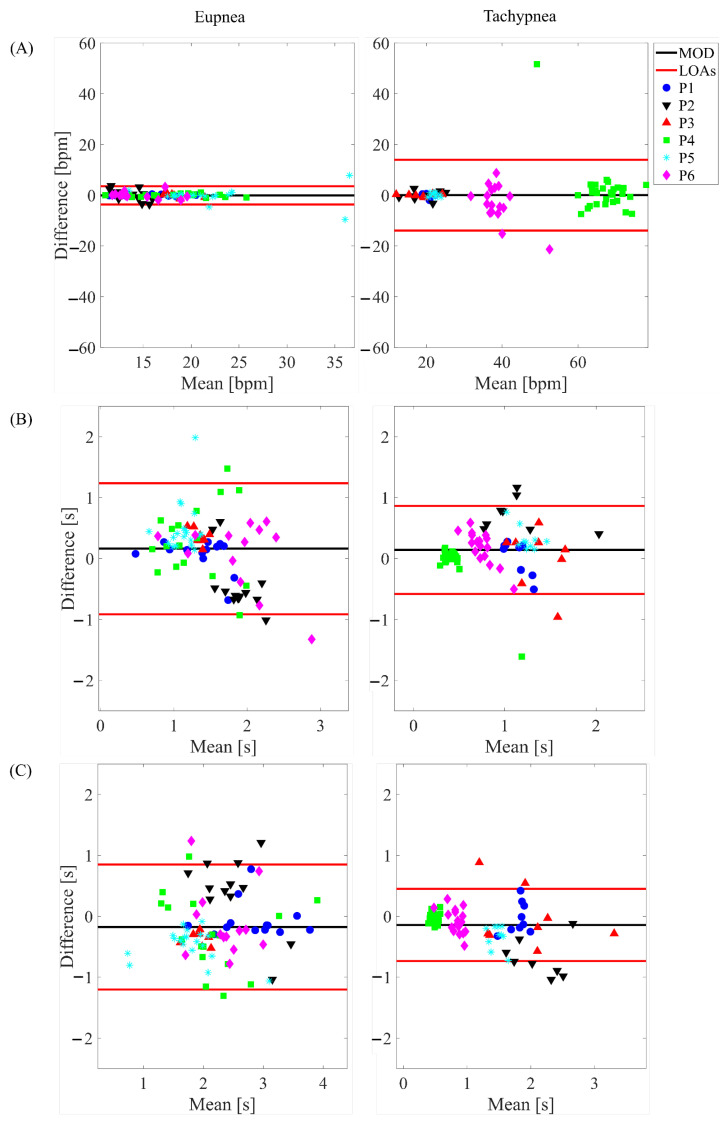
Bland-Altman plots depicting the estimated (**A**) $f_{r}$, (**B**) $t_{i}$ and (**C**) $t_{e}$ on the respiratory signal retrieved from the affected side in eupnea and tachypnea trials.

The BA plots depicted in Figure 6 show the results for the total respiratory signal, in Figure 7 for the healthy side respiratory signals, and in Figure 8 for the affected side respiratory signals. The obtained values of MOD, LOA amp, and MAE are reported in Table 3. Considering all the conditions, the most relevant differences between the WD and the gold standard were found for $f_{r}$. Indeed, the highest values in terms of both MAE and LOA amp (i.e., 3.24 bpm, and 27.90 bpm) were found in the tachypnea regime, especially when sensors of the affected side were used (aa).

## 4. Discussion and Conclusions

Physiological parameters monitoring may provide a valid support for evaluating the health status of a person even more, when other pathologies or complications entail major attention to the clinical picture. In post-stroke patients, the muscular impairment due to the stroke affects organs and muscles devoted to respiration causing abnormal or unusual breathing-related parameters values.

The gold standard technology for assessing the respiratory activity of these patients are OEPs. They allow an accurate analysis, but only in structured environments with qualified personnel. In addition, the high cost, time-consuming activity of preparing the patient for the therapy, and limited use in a structured environment, make OEPs’ use discouraging and/or sometimes uncomfortable for the patient. An alternative to OEPs are spirometers; however, they also require qualified personnel, provide discomfort to the patient due to the need of the mouthpiece, and do not allow chest wall compartments’ monitoring [48,49]. Basically, both these systems cannot be used for continuous monitoring of respiratory activities. A valid solution to overcome these hurdles is represented by wearable systems.

Wearables’ potentiality for monitoring respiratory activity has been exploited in various scenarios (e.g., sports science, occupational settings, and many clinical applications) [50,51], but their feasibility in post-stroke patients is still unexplored.

To this scope, in this study we investigate the capability of a multi-sensor WD based on piezoresistive sensors to monitor the respiratory parameters in six post-stroke patients, and we compared its performance with an OEP used as the gold standard.

We assessed the performance of the WD by estimating the respiratory frequency and the timing of the respiratory phase (i.e., $t_{i}$, and $t_{e}$) in eupnea and tachypnea trials. During both the respiratory regimes, the analyses were performed considering all the four sensors embedded within the wearable (to exploit information from the total chest wall), the two sensors placed on the healthy side (to use information from all the healthy side), and the two sensors placed on the affected side (to use information from all the plegic side). The WD showed good agreement with the gold standard in all the tested chest wall compartments and respiratory regimes in terms of mean respiratory frequency. This is testified by the low values of mean err and MAE (i.e., always lower than 0.35 bpm in eupnea, and always lower than 1.35 bpm in tachypnea). It is worth noting that the higher errors have been obtained considering the sensors placed in the affected side compartment, especially in tachypnea.

In addition, the WD showed good agreement with the gold standard also in terms of breath-by-breath respiratory frequency ($f_{r}$), $t_{i}$, and $t_{e}$ as testified by the low values of MOD and MAE. By comparing the agreement between the WD under the test and the gold standard and considering all the conditions (different respiratory regimes and sensors used to extract the parameters), the most relevant differences were found in the estimation of $f_{r}$. Indeed, as reported in Table 3, both MAE and LOA amp values strongly increase in the tachypnea regime, especially when sensors of the affected side were used. This might be due to the respiratory signal morphology, which affects the performance of the estimation. Indeed, the slight abnormalities in the respiratory signal can make the peak detection more challenging, causing an additional error. Probably, these abnormalities significantly affect the signals of the two sensors placed on the affected side. Conversely, when the analysis is performed either on all the sensors (total chest wall) or on the two sensors placed on the healthy side, the influence of these respiratory abnormalities is reduced. This speculation is fueled by the influence of the respiratory regime: the deterioration of the performance in the $f_{r}$ estimation under tachypnea may be motivated by the emphasized unnatural respiratory kinematics under respiratory effort, as reported in the literature [40,52]. Indeed, in [40], the respiratory volume of the paretic side demonstrated a significant reduction compared to the healthy one. Therefore, in demanding respiratory regimes (i.e., hyperventilation, tachypnea), the respiratory-related muscles are strongly affected by the muscular impairment causing an asymmetry between the healthy and paretic side volumes.

The literature demonstrated better results but were obtained on healthy subjects. In [28], a bias of 0.001 ± 0.453 s has been obtained for the respiratory period using a t-shirt instrumented by four inductive sensors, and [36], where the respiratory frequency and the respiratory period demonstrated a bias of −0.02 ± 1.04 bpm and 0.01 ± 0.19 s, using an FBG-based wearable device. In addition, in [53], inertial measurement units have been used to retrieve the respiratory parameters (i.e., $f_{r}$, $t_{i}$ and $t_{e}$) in supine and seated positions. Considering the seated position, $f_{r}$, $t_{i}$ and $t_{e}$ demonstrated biases of 0.44 ± 2.69 bpm, 0.06 ± 0.24 s, and 0.02 ± 0.28 s, respectively. In [31], piezoresistive sensors have been used to monitor the respiratory frequency in eupnea and tachypnea regimes, obtaining biases of 0.05 ± 1.97 bpm and 0.27 ± 11.27 bpm, respectively.

Although the results obtained in this pilot study on hemiplegic patients are slightly worse than those reported on healthy subjects, they still are promising and highlight the potentiality of the proposed WD on these patients. Moreover, the developed WD, thanks to its multi-sensor design, allows for monitoring the respiratory activity in the different chest wall compartments; this aspect was not taken for granted due to the challenging application. Indeed, considering that the WD retrieves the respiratory signals by measuring the strain provided by the breathing-related chest wall motion, abnormalities in this movement may cause the WD to fail in the measurement. Thus, it is of great interest to assess the feasibility of this device on this cohort of patients. In addition, the multi-sensor design paves the way to perform advanced analyses on other respiratory parameters associated to the impaired biomechanics. As a matter of fact, using the respiratory signals obtained for the compartments and applying the method proposed by Konno and Mead [54], the time shift between the various compartments or the contribution of each compartment to the total lung volume could be retrieved. Despite there being no survey focused on the preference by the patient in using the WD, far over the standard of care was performed, and each patient provided positive feedback on the wearability and comfortability of the proposed WD. Therefore, it entails that an additional effort can be made to further improve the device’s performance and make it suitable for clinical assessments. Due to the principle of work of the sensing elements and on their fixation on the elastic bands, the assessment of the cross-sensitivity between sensors positioned on the same band would be of great interest in the perspective to use this device to estimate the respiratory compartmental volumes. Of course, the obtained results have been gathered on a small population, but still represent the first step in using a wearable system for monitoring respiratory parameters in post-stroke patients. In addition, we foresee that the improvement of the peak detection algorithm in combination with a sensor fusion approach to maximize the signal quality may enable the use of this device for clinical evaluation and remote monitoring.

## Figures and Tables

**Table 1:** Patient’s anthropometric measurements, affected side, and plegia severity.

Patient ID	Age [year]	Height [m]	Mass [kg]	BMI [kg/m^2^]	Affected Side	Severity
1	62	1.70	77	26.6	left	32
2	46	1.61	50	19.3	right	33
3	64	1.74	99	32.7	right	43
4	33	1.68	54	19.1	left	55
5	55	1.68	49	17.4	right	50
6	43	1.65	75	27.6	left	34

**Table 2:** Err and MAE for the tt, hh, and aa comparisons.

Patient ID	Eupnea			Tachypnea		
	tt	hh	aa	tt	hh	aa
1	0.00	1.00	0.00	0.00	2.01	0.00
2	0.00	0.00	0.00	0.01	0.01	2.01
3	0.01	0.01	0.01	−0.01	−0.01	5.99
4	0.00	0.00	−1.00	−0.01	−0.01	−0.01
5	0.00	1.02	−1.01	0.00	0.00	0.00
6	0.00	0.00	0.00	0.01	0.01	0.01
**Mean**	0.00	0.34	−0.33	0.00	0.33	1.33
**MAE**	0.00	0.34	0.34	0.01	0.34	1.34

**Table 3:** MOD, LOA amp, and MAE values obtained for the performed BA analyses. The BA analyses were performed on the total (tt), healthy side (hh), and affected side (aa) respiratory signals.

	Eupnea			Tachypnea		
	**tt**	**hh**	**aa**	**tt**	**hh**	**aa**
$f_{r}$ **[bpm]**						
MOD	0.23	0.07	−0.04	0.03	−0.57	0.05
LOA amp	9.00	3.14	7.21	9.96	17.93	27.90
MAE	0.50	0.55	0.96	2.64	3.14	3.24
$t_{i}$ **[s]**						
MOD	0.37	0.36	0.16	0.18	0.16	0.14
LOA amp	1.47	1.96	2.15	0.91	1.16	1.44
MAE	0.42	0.49	0.46	0.23	0.25	0.27
$t_{e}$ **[s]**						
MOD	−0.41	−0.37	−0.18	−0.18	−0.15	−0.14
LOA amp	1.77	2.06	2.05	0.96	1.45	1.18
MAE	0.42	0.49	0.46	0.44	0.26	0.23

## Data Availability

The data presented in this study are available on request from the corresponding author. The data are not publicly available due to privacy reasons.

## Abbreviations

The following abbreviations are used in this manuscript:

OEP	Opto-electronic plethysmography
FBG	Fiber Bragg grating
WD	wearable device
PCB	Printed circuit board
mocap	motion capture system
Rc	Rib cage
AB	Abdomen
BMI	Body mass index
PSD	Power spectral density
MAE	Mean absolute error
bpm	Breaths-per-minute
BA	Bland-Altman
MOD	Mean of differneces
LOA	Limits of agreement
